# Supplementary material for: Association between Exposure to Ambient Air Pollution and Age-Related Cataract: A Nationwide Population-Based Retrospective Cohort Study
Source: Int J Environ Res Public Health. 2020 Dec 10;17(24):9231. doi: 10.3390/ijerph17249231 (PMC7763970; doi:10.3390/ijerph17249231)
Supplement: Supplementary file 1 [file ijerph-17-09231-s001.pdf]

Supplementary Table S1. The air pollutants and meteorological data in Korea from 2002 − 2015

Variables	Mean	SD	IQR	Percentiles				
				Minimum	25th	50th	75th	Maximum
Air pollution								
$PM_{2.5}$, μg/m^3	23.1	4.4	7.0	16.0	20.3	22.0	27.3	30.0
PM_{10}, μg/m^3	53.0	4.9	9.1	44.8	48.5	52.4	57.6	61.0
CO, 10 ppm	5.9	0.7	1.1	5.2	5.3	5.6	6.4	7.0
SO_2, ppb	5.7	0.5	0.7	4.7	5.3	5.7	6.0	6.4
NO_2, ppb	25.3	1.9	2.1	23.0	24.0	25.0	26.1	29.1
O_3, ppb	24.9	4.1	5.4	17.1	21.8	24.4	27.1	38.0
Annual weather conditions								
Average temperature, °C	12.8	0.6	1.1	12.0	12.2	12.9	13.3	13.6
Total rainfall, mm	1486	395	444	792	1320	1451	1764	2044
Average wind speed, m/s	2.48	0.24	0.3	2.0	2.4	2.45	2.7	2.8

Particulate matter <10 μm (PM_{10}), Sulfur dioxide (SO_2), Nitrogen dioxide (NO_2), Carbon monoxide (CO) and Ozone (O_3) were measured between 2002 − 2015. Particulate matter < 2.5 μm ($PM_{2.5}$) was measured in 2015.

Temperature, rainfall and wind speed were shown at Seoul (Lat.(N) 37˚34′, Long.(E) 126˚57′). Korea Meteorological Administration, Seoul, Korea
